# Difference-making factors for successful implementation of a multicomponent colorectal cancer screening program in rural clinics (SMARTER CRC)

**DOI:** 10.3389/fmed.2025.1522738

**Published:** 2025-06-25

**Authors:** Amanda F. Petrik, Brittany Badicke, Melinda M. Davis, Edward J. Miech, Jennifer Coury, Erin S. Kenzie, Jennifer L. Schneider, Robert Durr, Anna C. Edelmann, Anders Herreid-O’Neill, Emily Myers, Gloria D. Coronado

**Affiliations:** ^1^Kaiser Permanente Center for Health Research, Portland, OR, United States; ^2^Oregon Rural Practice-Based Research Network, Oregon Health & Science University, Portland, OR, United States; ^3^OHSU-PSU School of Public Health, Oregon Health & Science University, Portland, OR, United States; ^4^Department of Family Medicine, Oregon Health & Science University, Portland, OR, United States; ^5^Indiana University School of Medicine, Indianapolis, IN, United States; ^6^University of Arizona, Tucson, AZ, United States

**Keywords:** colorectal cancer, colorectal cancer screening, fit testing, implementation science, coincidence analysis (CNA)

## Abstract

**Introduction:**

Rural disparities in colorectal cancer (CRC) screening persist despite the availability of effective, evidence-based interventions. In this study, we aimed to understand what characteristics lead to success when implementing a multicomponent CRC screening intervention in rural primary care clinics in a pragmatic clinical trial (SMARTER CRC).

**Methods:**

We applied coincidence analysis to identify solution pathways that led to successful implementation during the first year of SMARTER CRC in intervention clinics. We assessed clinic success as high/low rates of fecal immunochemical testing (FIT) and overall CRC screening. Factors included in the analysis were collected through qualitative interviews, practice facilitation notes, and project datasets.

**Results:**

A total of 14 intervention clinics were included in our analysis. Post-intervention, overall clinic-level screening rates for CRC ranged from 12.6 to 22.0%, while FIT completion rates among patients who were mailed a kit ranged from 12.3 to 41.7%. Values for three factors perfectly distinguished between clinics with higher and lower CRC screening rates: clinics sending a pre-FIT introduction letter on their own, clinics having prior (or current) experience with CRC screening campaigns, and clinics changing the type of FIT they used. For FIT screening rates, two factors perfectly distinguished between clinics with higher and lower rates: clinics sending introduction letters on their own and clinic staff attending four or more health plan/clinic meetings.

**Discussion:**

Higher FIT and CRC screening rates were associated with clinics that were able to mail an introductory letter, had experience in CRC screening campaigns, did not change their FIT, and attended the health plan/clinic meetings. These clinic-level factors appear to be difference-makers to the successful implementation of a CRC screening program in rural settings.

## Introduction

Colorectal cancer (CRC) is the fourth most common type of cancer and the second leading cause of cancer deaths in the United States, representing approximately 8% of new cancer diagnosis and over 50,000 deaths in 2023 (1). Screening for CRC is highly effective in detecting cancer in early stages, achieving a 90% 5-year survival rate when found at the localized stage; however, between 2016 and 2020, approximately only one in three cancers were identified at this stage (2). More than half of CRC deaths can be prevented by screening and early detection, yet barriers persist at the patient, provider, and health system levels, with unique challenges in rural and frontier communities (3–6). Rural residents experience higher mortality from CRC than their urban counterparts due to persistent rural disparities in cancer screening and prevention (7). It is well-documented that these health disparities are often attributed to limited access to healthcare, inadequate health insurance, and higher poverty rates for rural Americans than their urban counterparts (8).

The implementation of mailed fecal immunochemical testing (FIT) and patient navigation programs can increase the uptake of CRC screening in clinical practices (9, 10). Prior research reports the effectiveness of CRC screening programs, including FIT screening and patient navigation in large health systems (10–13). While there has been an increase in the use of FIT as a first-line mechanism for CRC screening, substantial variation remains in implementation strategies and program adaptations when this evidence-based intervention is integrated into practice (14–19). Clinic and health plan partnered programs can increase the uptake of screening and follow-up through patient navigation; however, a better plan is needed to understand the key implementation factors for success (20, 21). Limited research has explored the factors associated with the successful implementation of multi-level programs to increase CRC screening in rural primary care settings (22).

This study examines features affecting the effectiveness of a multicomponent program of mailed FIT outreach and patient navigation to boost CRC screening in rural primary care. The SMARTER CRC study tested the implementation of a mailed FIT and patient navigation program in rural and frontier clinics using a multi-level clinic and health plan partnered approach (23). Implementation was supported by study practice facilitators trained in the intervention (24). We used data from the SMARTER CRC study to understand which clinic- or community-level characteristics explained implementation success. We aimed to understand combinations of implementation-related activities and clinic conditions that consistently distinguished intervention clinic sites with higher overall CRC screening rates compared to those with lower ones, as well as FIT return rates from mailed outreach. These findings can be used more broadly in the planning, adaptation, and implementation of mailed FIT programs in rural settings.

## Methods

### Study setting

SMARTER CRC is a pragmatic implementation trial partnering with Medicaid health plans and rural primary care clinics in Oregon to support the implementation of a mailed FIT outreach and patient navigation program (23). This study was conducted as part of the National Cancer Institute-funded Accelerating Colorectal Cancer Screening and Follow-up through Implementation Science (ACCSIS) Program. The overall aim of ACCSIS is to conduct multi-site, coordinated, transdisciplinary research to evaluate and improve CRC screening processes using implementation science. This study was approved by the Oregon Health & Science University Institutional Review Board (STUDY00020681); individual consent was not required from clinical patients receiving the intervention as it was determined to be a pragmatic extension of clinical practice. Qualitative interview participants verbally consented to the interviews.

Details of the SMARTER CRC design and outcomes have been described in a previous study (23, 30). In brief, intervention clinics were randomly selected to implement the mailed FIT outreach and patient navigation program during the first year of the trial, while the remaining clinics continued with usual care. Medicaid health plans affiliated with intervention clinics generated the lists of patients due for CRC screening and provided them to the clinics. Clinic staff reviewed the list and removed any patients who were ineligible for screening or had not yet established care. The revised lists were sent to a mailed vendor who mailed patients’ FITs and clinics and/or health plans sent FIT reminders. At each clinic, medical assistants or other patient support staff received training for patient navigation. Patient navigators (usually medical assistants or outreach staff) then provided navigation support through phone calls to patients with abnormal FIT results to complete a colonoscopy. Intervention clinics received practice facilitation as an implementation strategy. Practice facilitators are individuals trained to support clinical practices in capacity building and evidence-based intervention implementation (25, 26). Practice facilitators supported the navigators throughout the project; however, the implementation varied across clinics. For example, some clinics mailed introductory letters on their own, some opted to attend monthly meetings with clinics and health plans, and others opted to change their FIT types.

Within 28 randomized clinics, eligible patients were identified, and the intervention was implemented over 1 year, ranging from May 2021 to June 2022 (23). In this analysis, only data from the Year 1 (*N* = 14) intervention clinics are included.

### Study outcomes

Coincidence Analysis (CNA) is a configurational comparative method that enables the analysis of clinical, community, intervention, and implementation components that lead to implementation success (27).

CNA focused on two research questions from the Year 1 outcomes (main outcomes):

1. Which combinations of implementation-related activities and clinic conditions consistently distinguished sites with higher CRC screening rates from those with lower CRC screening rates?
2. Which combinations of implementation-related activities and clinical conditions consistently distinguished sites with higher FIT return rates from those with lower FIT return rates?

Outcomes for the CNA include overall CRC screening rates (high/low) and FIT return rates (high/low). CRC screening rates are calculated from the overall eligible population, while the FIT return rates are calculated from the population who were mailed kits.

### Intervention and measures

Data were generated from clinic intake surveys, practice facilitation field notes, qualitative interviews, claims data, and data logged by the clinics in the REDCap research data capture tool during program implementation (28, 29). First, data were collected through a Baseline Intake Survey that was distributed to the clinics by the research team. This survey collected information concerning clinic activities, including prior CRC screening programs, FIT, CRC screening rates, and staffing. Data were also collected through practice facilitation notes and activities. The practice facilitators documented scheduled and *ad hoc* interactions, level of engagement, progression of study activities, concerns about the ability to progress, facilitator-needed supports to help clinical practices, and adaptations through contemporaneous contact logs entered in structured forms in REDCap.

Qualitative interviews were conducted with at least one staff member at each clinic (e.g., practice managers, clinical informatics/EHR specialists, quality improvement specialists, medical assistants, providers) at baseline and included information on the clinic and health plan relationship, clinic characteristics, and details about the clinical experience. The interviews were recorded, professionally transcribed, and validated against source audio for accuracy and stored in ATLAS.ti for management. Questions related to specific study activities and site characteristics were identified within the clinic baseline interviews, and clinic answers were categorized into yes/no or high/medium/low variables for the analysis. Quantitative data included data collected from the Medicaid health plans (i.e., claims data) and data collected from clinics and stored in project datasets (REDCap).

Data were collected from the above sources into a single dataset to determine which implementation-related activities for the 14 intervention sites together might explain the outcomes (Table 1). The original dataset had 58 potential explanatory variables, with two different outcomes of interest: overall CRC screening rates (any modality) and FIT return rates.

**Table 1:** Implementation factors and clinic conditions included in CNA model.

Source	Description
Quantitative data	
Clinic characteristics	Federal designation, network structure, EHR, lab
Community data	Income to poverty level, % of adults with less than high school education, poverty status, total population, % of non-Hispanic whites, % of female-headed households, % households receiving public assistance, % of men who are unemployed
Rurality	Oregon rural health designation, Rural–Urban Commuting Area Codes (RUCA)
Clinic survey characteristics	CRC champion, prompt calls, FIT characteristics (i.e., where FITs processed, FIT test), reminders (i.e., messages to patients, reminder texts, reminder calls), navigation, scrub, clinician attitudes on CRC screening, clinic supports, clinic priorities, leadership characteristics
Health plan characteristics	Health plans, mailing characteristics, text reminders
Qualitative data	
Clinic health plan relationship	Research staff perception of relationship of clinic to health plans, clinic perception of health plan-clinic relationship, clinic perception of level of health plan support received
Clinic characteristics	Staffing issues prior to implementation, attitude toward FIT, prior disruptions (new EHR or increases in pop serving), attitude to Mailed FIT, Training on CRC screening (ongoing training of MAs, staff, providers, etc. on workflow or choices)
Clinic experience	Involvement in awareness campaigns, prior FIT Mailing, other current CRC campaigns past or current
Practice facilitation acquired data	
Project characteristics	Health plan supplied FIT vs. clinic supplied FIT, clinic choice to scrub the patient list, clinic choice to send an introduction letter, introduction letter sent by health plan, implementation of clinic-level prompt calls, implementation of health plan-delivered prompt calls, implementation of clinic-delivered reminder calls, implementation of health plan-delivered reminder calls
Engagement	Monthly health plan-clinic meeting attendance, patient navigation training attendance, level of engagement in study activities (beginning, mid-point, and end of Year 1)
Disruptions	Disruption in main point of contact
Adaptations	Clinic-level adaptation where there was a mention of significant adaptation in REDCap

### Analysis

The R package “cna” was used to analyze the dataset. RStudio, R, and Microsoft Excel were also used to support the analysis. The site-level overall CRC screening rates were calculated by the number of patients completing any CRC screening out of the intention-to-treat (ITT) population (30). The overall CRC screening values after 1 year for the 14 sites ranged from 12.6 to 22%, with a median value of 19.1% and a full 1.5-point difference between the two closest outcome values on either side of the meridian value (18.4% vs. 19.9%). For the analysis, sites with overall CRC values above the median were categorized as sites with higher rates and assigned an outcome value of 1; sites with overall CRC values below the median were categorized as sites with lower rates and assigned an outcome value of 0.

The site-level FIT return rates were calculated as the number of patients who returned a FIT divided by the number of patients that were mailed a FIT. The FIT outcome values ranged from 12.3 to 41.7%. Given the relative tight clustering of values between 18.9 and 21.4% for six sites, followed by a full 2-point gap until the next highest value of 23.7%, the analysis categorized FIT values of ≥23% as sites with higher FIT rates and assigned an outcome value of 1 and FIT values of <23% were categorized as sites with lower FIT rates and assigned an outcome value of 0.

To prepare the dataset for analysis with CNA, continuous variables were recoded as categorical factors, and missing values were temporarily assigned a dummy value to keep them from dropping out of the analysis.

To achieve data reduction, an exploratory data analysis was conducted on the entire dataset to inform the selection of a smaller subset of candidate factors for use in subsequent model development. Specifically, the “minimally sufficient conditions” (i.e., “msc”) function from the R package “cna” was used to search across all 61 cases and all process and context factors (with process factors assessed by three rates across all five time points) to identify redundancy-free configurations of specific conditions with specifically strong connections to the outcome of interest (19, 31–39). This exhaustive process considered every possible one-, two-, and three-condition configuration present in the dataset, assessed each configuration against a prespecified consistency threshold, and retained configurations that meet the consistency threshold.

During this exploratory data analysis, the “msc” function was run multiple times at different consistency levels (95, 90, 85, 80, and 75%) to compare output at different thresholds (32, 33). The study team reviewed the output to identify a small number of “best of class” configurations that met all of the following criteria: (1) the highest coverage score within configurations of identical length (i.e., the “complexity level”); (2) having a significant difference between top-scoring coverage configuration and its next-nearest neighbor within the same complexity level; (3) substantive plausibility; (4) and relevance to our research question.

We then iterated the model using the subset of factors represented by these best-of-class configurations. Using this bottom-up approach, the original dataset was inductively analyzed in its entirety, drawing upon substantive knowledge when interpreting the mathematical output generated by the msc routine, and ultimately identified a subset of candidate factors for model development during the next step of the CNA.

During model development, the goal was to develop overall models that met all of the following criteria: scores of >80% for both consistency and coverage; inclusion of the same factors (taking on different values) to explain both the presence and the absence of the outcome; alignment with theory and prior knowledge; inclusion of at least one program-related factor; relevance to our research question; and absence of model ambiguity.

## Results

Of the 14 clinics in the first year of SMARTER CRC, CRC screening rates among the identified eligible population ranged from 12.6 to 22.0%, and FIT return rates among patients who were mailed a FIT ranged from 12.3 to 41.7% (Table 2). The number of eligible patients ranged from 32 to 1,154, and the number of patients who were mailed a FIT ranged from 14 to 579 across these clinical sites.

**Table 2:** Clinical characteristics and outcomes.

Clinic	Number of eligible patients	CRC screening rate*	Mailed FIT	FIT screening rate**	CRC screening rate at randomization	Health plan (1, 2, 3)	RUCA^†^ code
Clinic 13	91	22.0%	44	29.5%	52%	2	7
Clinic 5	32	21.9%	14	21.4%	60%	2	4
Clinic 10	183	21.7%	114	24.3%	55%	2	4
Clinic 12	83	21.7%	24	41.7%	Unknown	2	4
Clinic 8	159	21.4%	113	23.9%	39%	2	7
Clinic 9	45	20.0%	35	20.0%	40%	1	5
Clinic 11	256	19.9%	216	18.5%	50%	1	7
Clinic 3	49	18.4%	30	13.3%	52%	1	5
Clinic 14	47	17.0%	31	19.4%	31%	1	4
Clinic 4	39	15.4%	29	13.8%	41%	1	5
Clinic 7	145	15.2%	96	18.8%	50%	2	10
Clinic 6	106	15.1%	73	12.3%	32%	1	4
Clinic 1	1,154	13.3%	579	16.4%	18%	3	4
Clinic 2	224	12.6%	91	18.9%	33%	1	4

Green color shows high outcome; orange color shows low outcome. *% of all eligible patients; **% of patients mailed FIT.

† Rural–Urban Commuting Area Codes, 1 is metropolitan, 10 is rural.

The analysis results presented in Table 3 focused on the research question: *Which combinations of implementation-related activities and clinical conditions consistently distinguished sites with higher CRC screening rates from those with lower CRC screening rates?* The final models for CRC screening featured just three factors: clinics that were mailed pre-FIT introduction letters on their own; clinics that had past or current CRC screening campaigns; and clinics that did not change their FIT type. The positive model (CRC screening rate ≥ 19.1% = 1) featured two solution paths (i.e., two different paths to higher overall CRC screening rates). Solution Path 1 included clinics that chose to send out an introduction letter on their own, and Solution Path 2 included a combination of two conditions: clinic experience with past or current CRC campaigns (“other CRC campaign”) together with no clinic-level adaptation of FIT type (they did not change their FIT mid-project). The negative model for clinics with lower CRC screening values consisted of two solution pathways featuring the same three factors, but with different values. Solution Path 1 for the negative model involved the bundle of clinics not sending out the introduction letter on their own, together with no experience with other CRC campaigns. Solution Path 2 for the negative model involved clinics changing their FIT type. The model for the presence of the CRC screening outcome and the model for the absence of the outcome had perfect scores for consistency (7/7, 100%) and coverage (7/7, 100%). Consistency refers to how often clinics identified by the model had the higher screening rate present, while coverage accounts for the percentage of clinics with higher screening rates explained by the model. The same three factors perfectly distinguished between the clinics with higher and lower CRC screening rates.

**Table 3:** CNA analysis for CRC screening outcomes.

Clinic	CRC screening rate		Did the clinic mail out introduction letters?	Other CRC campaigns, past or current	Clinic adaptation, FIT type
	CRC screening rate ≥ 19.1%*		Solution path 1	Solution path 2	
Clinic 5	21.9%		1	0	0
Clinic 12	21.7%		1	0	0
Clinic 8	21.4%		1	0	0
Clinic 13	22.0%		1	1	0
Clinic 10	21.7%		1	1	0
Clinic 9	20.0%		0	1	0
Clinic 11	19.9%		0	1	0
	Overall model scores	Consistency	100% (7/7)		
		Coverage	100% (7/7)		
	CRC screening rate < 19.1%*		Solution path 1	Solution path 2	
Clinic 3	18.4%		0	Missing	1
Clinic 4	15.4%		0	Missing	1
Clinic 7	15.2%		0	1	1
Clinic 14	17.0%		0	0	1
Clinic 6	15.1%		0	0	0
Clinic 1	13.3%		0	0	0
Clinic 2	12.6%		0	0	0
	Overall model scores	Consistency	100% (7/7)		
		Coverage	100% (7/7)		

*The median value of overall CRC outcome values is 19.1%. Green color shows high outcome; dark orange color shows low outcome. Solution path shading (violet and orange) is used to highlight solutions.

The final models for higher vs. lower FIT return rates consisted of only two factors: whether or not clinics decided to send out introduction letters on their own and whether or not clinic staff attended four or more health plan/clinic meetings (Table 4). The positive model (FIT screening rate ≥ 23%) comprised a single solution pathway: the joint presence of sending out introduction letters on their own together with clinic staff attending four or more health plan/clinic meetings. The absence of either of these two factors was sufficient for lower FIT return rates. The positive model achieved perfect scores for both consistency (4/4, 100%) and coverage (4/4, 100%), as did the negative model, which also demonstrated both consistency (10/10, 100%) and coverage (10/10, 100%). There was modest model ambiguity in the results, in that a second, different factor was also identified as a candidate for both the positive and negative models for FIT return rates: whether the clinic reported navigating at least one patient with an abnormal FIT result. For the positive model, navigating at least one patient for an abnormal FIT result and deciding to send out introduction letters on their own independently accounted for all four clinics with higher FIT return rates, whereas the absence of either factor was sufficient for lower FIT screening rates. This alternative positive model had perfect scores for both consistency (4/4, 100%) and coverage (4/4, 100%), as did this alternative negative model for both consistency (10/10, 100%) and coverage (10/10, 100%). We ultimately selected “clinic staff attending four or more health plan/clinic meetings” as the second factor in our preferred models based on theoretical and practical grounds (which we address further in the “Discussion” section).

**Table 4:** CNA analysis for FIT screening outcome.

Fit screening rate ≥ 23%*			Solution path 1	
Clinic	FIT screening rate		Did the clinic mail out introduction letters?	Did the clinic attend 4 + project meetings?
Clinic 12	41.7%		1	1
Clinic 13	29.5%		1	1
Clinic 10	24.3%		1	1
Clinic 8	23.9%		1	1
	Overall model scores	Consistency	100% (4/4)	
		Coverage	100% (4/4)	
Fit screening rate < 23%			Solution path 1	Solution path 2
Clinic 5	21.4%		1	0
Clinic 9	20.0%		0	0
Clinic 2	18.9%		0	0
Clinic 6	12.3%		0	0
Clinic 14	19.4%		0	1
Clinic 7	18.8%		0	1
Clinic 11	18.5%		0	1
Clinic 1	16.4%		0	1
Clinic 4	13.8%		0	1
Clinic 3	13.3%		0	1
	Overall model scores	Consistency	100% (10/10)	
		Coverage	100% (10/10)	

*The median value of FIT Screening Rate values is 23%. Green color shows high outcome; dark orange color shows low outcome. Solution path shading (orange) is used to highlight solutions.

## Discussion

Higher FIT return and CRC screening rates were associated with clinics that were able to mail an introductory letter, had experience in CRC screening campaigns, did not need to change their FIT types, and attended the health plan-clinic meetings. Because SMARTER CRC was a pragmatic trial, each health plan approached program implementation differently, depending on their organizational context. While many clinical and implementation characteristics were assessed, the analysis identified success based on implementation choices and prior implementation experience. These approaches could be successfully employed across many settings and populations. Consistent engagement and participation in the project are crucial for implementation success.

Prior studies conducted by members of this team used CNA to understand implementation characteristics that improved the performance of mailed FIT programs. For example, one study found that involving support staff improved FIT completion rates in community clinics, as evidenced by higher screening rates following the implementation of a centralized mailed FIT program in clinics that had increased back-or front-office staff, had staff help patients resolve barriers to CRC screening, or handed out FITs while educating patients (14). Another study found that centralized implementation teams with dedicated staffing time and the mailing of an introductory letter led to the implementation success of increased FIT mailings (31). A final study using CNA found that health systems that used multiple adaptations to a screening program had higher screening rates, but no single adaptation clearly led to higher screening rates (19).

Regarding any CRC screening, our findings in this project suggest that the implementation strategies most closely associated with success included clinics choosing to send the introduction letter on their own, those participating in a past or current other CRC screening campaign, and maintaining clinics’ FIT type. Regarding FIT return rates, our findings suggest that the implementation strategies most predictive of success were clinics choosing to send their own introduction letter and attending four or more health plan-clinic meetings. Health plan 2 utilized a third-party full-service vendor with a specific FIT type and non-customizable materials. Health plans 1 and 3 were more customizable, allowing each clinic to choose which FIT to use and to customize materials to include clinic and health plan branding. For health plan 1, the clinic could choose to use the health plan FIT with central processing. Health plans 1 and 3 offered clinics to process FITs using their typical process.

Notably, clinical practices choosing to send the introduction letter on their own were a key difference maker for both CRC screening and FIT return outcomes. Furthermore, this intervention component has been predictive of screening success in prior studies (31). However, not all clinics were given the option of sending their own letter. One health plan partnered with a third-party vendor with vendor-branded materials, and clinics were given the option to send their own clinic-branded letter; many of these clinics chose to also send their own clinic-branded letter, leading to the patient receiving two notification letters. For the others, the health plans and clinics collaborated to produce co-branded materials that were mailed by the health plan. This intervention component may also be a clinical indicator of fidelity to the project and the recommended processes.

In this study, clinics collaborated with their Medicaid health plans to implement program components, and not all implementation elements were decided at the clinic level. This program included the mailing of a customized introductory letter that emphasized the importance of CRC screening and this easy, at-home testing option. The ability to execute the program was largely identified as high for clinics that had prior experience with CRC screening campaigns. When the introductory letter was sent from the clinic, the messaging was customized to the patient population and branded with clinic materials, potentially creating a greater sense of trust among the patient recipients. Successful clinics did not need to adapt the FIT they were using. Finally, monthly health plan-clinic meetings, led by the research team, served as a platform to share information about the program broadly and ask health plans and clinics to share their progress, successes, and lessons learned. The clinics that attended the meetings regularly experienced a greater success rate on their screening program, potentially indicating a clinic-level indicator of fidelity.

It should be noted that Rural–Urban Commuting Area Codes (RUCA) for rural designation failed to emerge as difference-makers in implementation success, although RUCA explained one case in the CNA. RUCA codes categorize geographical areas (zip) by population size (40). The RUCA codes indicated clinics were located in the micropolitan, rural and frontier areas. Clinics in rural and frontier areas are small enough to have easily changeable screening rates but have struggled with making practice changes and changing patient behavior. The complexity of rural and frontier clinics will need to be further studied to better understand implementation successes and challenges.

As mentioned in the “Results” section, some model ambiguity emerged due to a second, different factor identified as a candidate in both the positive and negative models for FIT return rates: whether the clinic reported navigating at least one patient for an abnormal FIT result. We ultimately selected the models featuring “clinic staff attending four or more health plan-clinic meetings” instead of this alternative second factor, as it demonstrated their investment in the intervention and could potentially reflect a broader implementation of CRC screening in their clinics. The clinics were willing to take the time to attend the meetings. The meetings themselves provided substantial advice regarding how to best implement the intervention components and offered an opportunity to workshop problems that arose during the roll-out of activities. Regardless, the dedication to conducting navigation may be an indicator of fidelity to the program as well as the navigator had to follow research processes to log navigation activities.

### Implications

With persistent disparities in CRC screening, these results point to the importance of engaging clinical practices and health plans in screening outreach campaigns to reduce the urban–rural practice gap. Our results indicate that clinical practices need a starting point to implement programs based on evidence-based strategies. Developing new screening programs, or evaluating prior screening programs and current testing processes, could be an intervention strategy for increasing implementation success.

It is important to create opportunities for collaboration between clinics and health plans (i.e., collaborative cross-sector meetings) to support program implementation. A key factor for success was the regular engagement with participating clinical practices, most notably during the monthly health plan and clinic meetings. This regular meeting cadence enables clinical practices to maintain momentum in their efforts and holds them accountable, as they are required to report on their current progress and any challenges they are experiencing. We found that this was a key component for clinical practices to maintain fidelity to the program.

## Limitations

This study is not without its limitations. First, given that the intervention occurred in rural and frontier primary care settings, the population sample size was inherently small. The threshold for clinical practices to engage in the study required at least 30 patients who met CRC screening eligibility criteria. It was not expected that all eligible patients would screen; therefore, the results were expected to encompass a small sample. Second, the intervention occurred during the height of the COVID-19 pandemic. Although a mailed screening outreach program has its benefits during a time when in-person interactions are discouraged, a majority of the primary care workforce was pulled to respond to the pandemic, limiting some staff capacity to fully engage in the programmatic activities (41).

### Future studies

It will be important for future research to continue exploring the complexity and nuances of the rural primary care environment, which includes collaborating with clinical practices that have not engaged in CRC screening programs to build the knowledge base of clinic-led CRC screening program implementation.

## Data Availability

The raw data supporting the conclusions of this article will be made available by the authors, without undue reservation.
